# Embryology of two mycoheterotrophic orchid species, *Gastrodia elata* and *Gastrodia nantoensis*: ovule and embryo development

**DOI:** 10.1186/s40529-016-0137-7

**Published:** 2016-08-08

**Authors:** Yuan-Yuan Li, Xiao-Mei Chen, Shun-Xing Guo, Yung-I Lee

**Affiliations:** 1 Institute of Medicinal Plant Development, Chinese Academy of Medical Sciences & Peking Union Medical College, Beijing, 100193 People’s Republic of China; 2 Biology Department, National Museum of Natural Science, No 1, Kuan-Chien Rd, Taichung, 40453 Taiwan; 3 Department of Life Sciences, National Chung Hsing University, Taichung, 40227 Taiwan

**Keywords:** Ovule, Embryo, Mycoheterotrophic orchids, *Gastrodia*, Suspensor

## Abstract

**Background:**

*Gastrodia elata*, a famous herbal medicine, has been received great attention on its treatments of headache, vertigo and epilepsy. *Gastrodia nantoensis* is a newly described species from central Taiwan with potential medicinal value. *Gastrodia* species are fully mycoheterotrophic orchids, and the courses of their seed development are more rapid as compared to the chlorophyllous orchids. A better understanding of their reproductive biology would provide insights into the propagation and conservation of the mycoheterotrophic orchid species.

**Results:**

Based on the histological and histochemical investigations, we observed some notable features in ovule and embryo development. First, only the archesporial cell and/or megasporocyte are present within their ovaries at the time of anthesis. Second, their suspensors consist of a single cell and their mature embryos consist of a gradient of small to large cells. Nile red staining of a globular embryo reveals the presence of cuticular material in the surface wall of embryo proper and the lateral walls of suspensor cell, indicating that the basal wall of suspensor cell is the major route for nutrient supply from maternal tissues to embryo proper. Third, their seed coats are derived from a single integument, and lignin but not cuticular material is present in the outer most layer of seed coat and persists through seed maturation.

**Conclusions:**

The faster seed development of *Gastrodia* species is due to the speedy courses of ovule and embryo development. In the mature seeds, the presence of a differentiated apical zone in embryo proper suggests the easy-to-germinate character. This study provides basic knowledge for further molecular studies on embryo development and symbiotic germination of *Gastrodia* species.

## Background

The tiny orchid seed has a rudimentary embryo and lacks endosperm; therefore, the germination of orchid seed requires the mycorrhizal association, which supplies nutrients for the germinating seed until the seedling differentiates green leaves and becomes autotrophic (Rasmussen 1995). Although a large amount of orchids are chlorophyllous, some orchids remain achlorophyllous and reply on their mycorrhizal partners for nutrient supplies throughout the entire life cycle, and they are known as mycoheterotrophic plants (Leake 1994; Merckx 2013; Lee et al. 2015). For many fully mycoheterotrophic orchids, their growth patterns are unique. They stay underground year-round, but after sprouting, the phases of blooming and then fruit setting complete within a few weeks. The course of seed development is more rapid than chlorophyllous orchids (Afzelius 1954; Arekal and Karanth 1981).

The genus *Gastrodia* comprises more than 50 species, distributing mainly in Asia and Oceania regions (Hsu 2008). All *Gastrodia* species are fully mycoheterotrophic, and they are considered as the largest mycoheterotrophic genus in Orchidaceae. *Gastrodia elata*, a representative *Gastrodia* species, is an important traditional Chinese medicine (known as Tian Ma) for treatment of headache, vertigo and epilepsy (Xu and Guo 2000). Besides *G. elata*, there are more than 20 *Gastrodia* species native in Taiwan (Hsu and Kuo 2010). Some of them may have medicinal values and are under the threats of over-collection for the medicinal market. *Gastrodia. nantoensis* is a newly described endemic species with only a few known populations in Taiwan. As compared to the growth of stalk of *G. elata*, the growth of stalk of *G. nantoensis* is quite different. *G. nantoensis* has a very short stalk at the time of anthesis, and the further elongation of stalk is triggered by pollination. Although the structure of *G. elata* embryology has been documented in previous studies by the line drawing or paraffin sections (Kusano 1915; Abe 1976; Liang 1981, 1984), information concerning the development of ovule and embryo of *G. nantoensis* is lacking. For the commercial production and the conservation works of *Gastrodia* species, information concerning its reproductive biology is essential. Basic knowledge of seed development will assist in the improvement of artificial propagation, as demonstrated in our previous studies of *Cypripedium formosanum* (Lee et al. 2005) and *Cypripedium macranthos* (Zhang et al. 2013).

The objectives of this study are to document the key anatomical events in the embryogenesis of two *Gastrodia* species during the courses of megasporogenesis, megagametogenesis, fertilization and seed maturity. In this study, we used the resin-embedded sections, providing high resolution, light microscopic interpretations of various developmental stages. The observations would shed light on the embryogenesis of *Gastrodia* species and add to the current literature.

## Methods

### Plant materials

Plants of *G. elata* were cultivated in the greenhouse at Institute of Medicinal Plant Development, Chinese Academy of Medical Sciences & Peking Union Medical College, Beijing, China, while plants in a natural population of *G. nantoensis* in the bamboo forest located at Nantou County, Taiwan were selected for this study. Anthesis of *G. elata* occurred from April to May, and anthesis of *G. nantoensis* occurred from September to October. To ensure a good fruit set and seed quantity, the flowers were hand-pollinated. Developing ovaries and fruits were harvested at regular intervals after pollination. Approximately 30 developing ovaries and fruits of each species were gathered for this study.

### Light microscopy and histochemical observations

Development ovaries and capsules were fixed with 2 % paraformaldehyde and 2.5 % glutaraldehyde in 0.1 M phosphate buffer, pH 6.8, at 4 °C overnight. After fixation, the samples were dehydrated using an ethanol series, and embedded in Technovit 7100 (Kulzer & Co., Germany) as described by Yeung and Chan (2015). Serial, 3 µm-thick sections were cut with glass knives using a Reichert-Jung 2040 Autocut rotary microtome. These sections were stained with Periodic acid–Schiff’s reaction for total insoluble carbohydrates, and counterstained with either 0.05 % (w/v) toluidine blue O (TBO) in benzoate buffer for general histology or 1 % (w/v) amido black 10B in 7 % acetic acid for protein (Yeung 1984). The presence of cuticular material was detected using Nile red as detailed in Lee et al. (2006). The sections were stained with 1 μg ml-1 of Nile red (Sigma Chemical Co., St. Louis, Mo.) for 5 min, briefly washed in distilled water for 1 min, and mounted in a solution containing 0.1 % n-propyl gallate (Sigma Chemical Co.), an antifading compound. The fluorescence signal was examined using an epifluorescence microscope (Axioskop 2, Carl Zeiss AG) equipped with the Zeiss filter set 15 (546/12 nm excitation filter and 590 emission barrier filter). These sections were viewed and the images were captured digitally using a CCD camera attached to the light microscope.

## Results and discussion

**Fig. 1:**
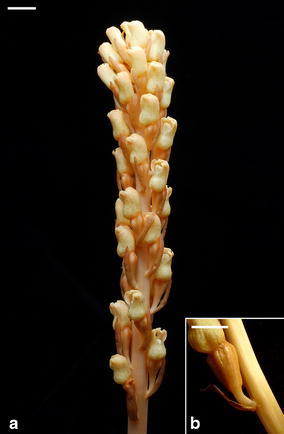
The flower and developing fruits of *G. elata*. **a** The stalk has elongated at the time of anthesis. *Scale bar* 1 cm. **b** The developing fruit at 6 DAP. *Scale bar* 1 cm

**Fig. 2:**
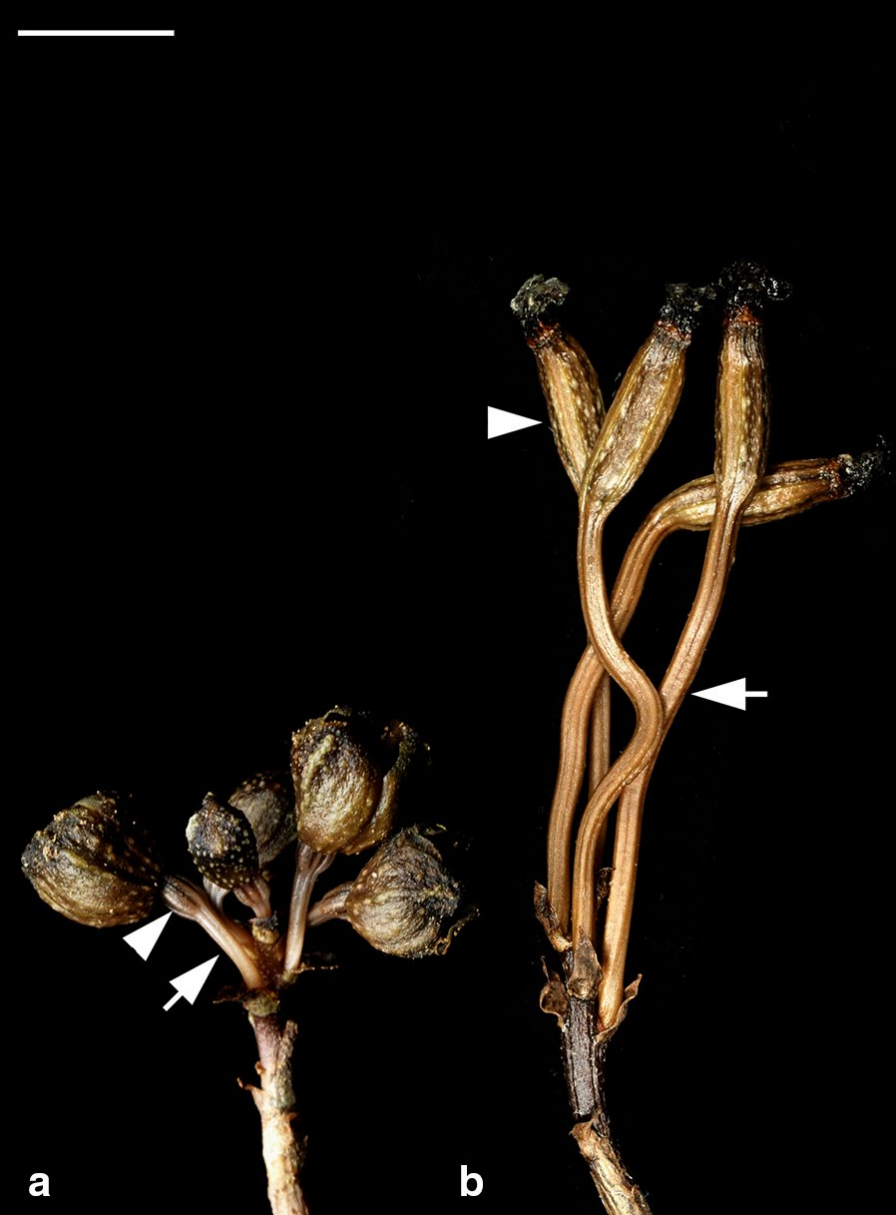
The flowers and developing fruits of *G. nantoensis*. **a** The stalks (*arrow*) are short and the fruits are small (*arrowhead*) at the time of anthesis. *Scale bar* 1 cm. **b** At 6 DAP, the stalks start to elongate rapidly (*arrow*) and the fruits have enlarged (*arrowhead*). *Scale bar* 1 cm

**Fig. 3:**
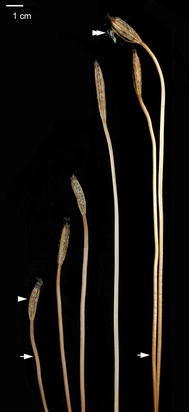
The elongating stalks and developing fruits of various stages of *G. nantoensis*. After 20 DAP, the seeds (*double arrowhead*) have matured and released from the split fruit. *Scale bar* 1 cm

The major post-pollination developmental events that occurred in the developing fruits of two *Gastrodia* species are summarized in Table 1. Both of them have similar patterns in ovule and embryo developments. The seed maturation of *G. elata* and *G. nantoensis* only took 16 and 20 days after pollination (DAP), respectively. In several mycoheterotrophic orchid species, the above-ground part remained for a few weeks, and then disappeared (Arekal and Karanth 1981; Lin 1977). Accordingly, the seed development of mycoheterotrophic orchid species was usually faster than most orchid species. As compared to the growth of stalk of *G. elata*, the growth of stalk of *G. nantoensis* was quite different. The stalk of *G. elata* had elongated before anthesis, while the stalk of *G. nantoensis* remained short at the time of anthesis (Figs. 1, 2). It was noticeable that the stalks of *G. nantoensis* started to elongate rapidly by 6 DAP. At maturity, the seeds were released from the split capsule and the stalk could reach to more than 40 cm in length (Fig. 3). In *G. nantoensis*, the rapid elongation of stalk triggered by pollination was helpful for the wind dispersal of seeds to long distance.

**Table 1:** Major microscopic structural events in the developing fruits of *G. elata* and *G. nantoensis*

Developmental stage	Days after pollination	
	*G. elata*	*G. nantoensis*
Archesporial cell and megasporocyte	0	0
Megasporogenesis and megagametogenesis	2	2–4
Mature embryo sac	4	6
Fertilization and zygote	6	8
Proembryo	8	12
Globular embryo	10	14–16
Late globular embryo	12–14	18
Mature seed	16	20

### Ovule development

**Fig. 4:**
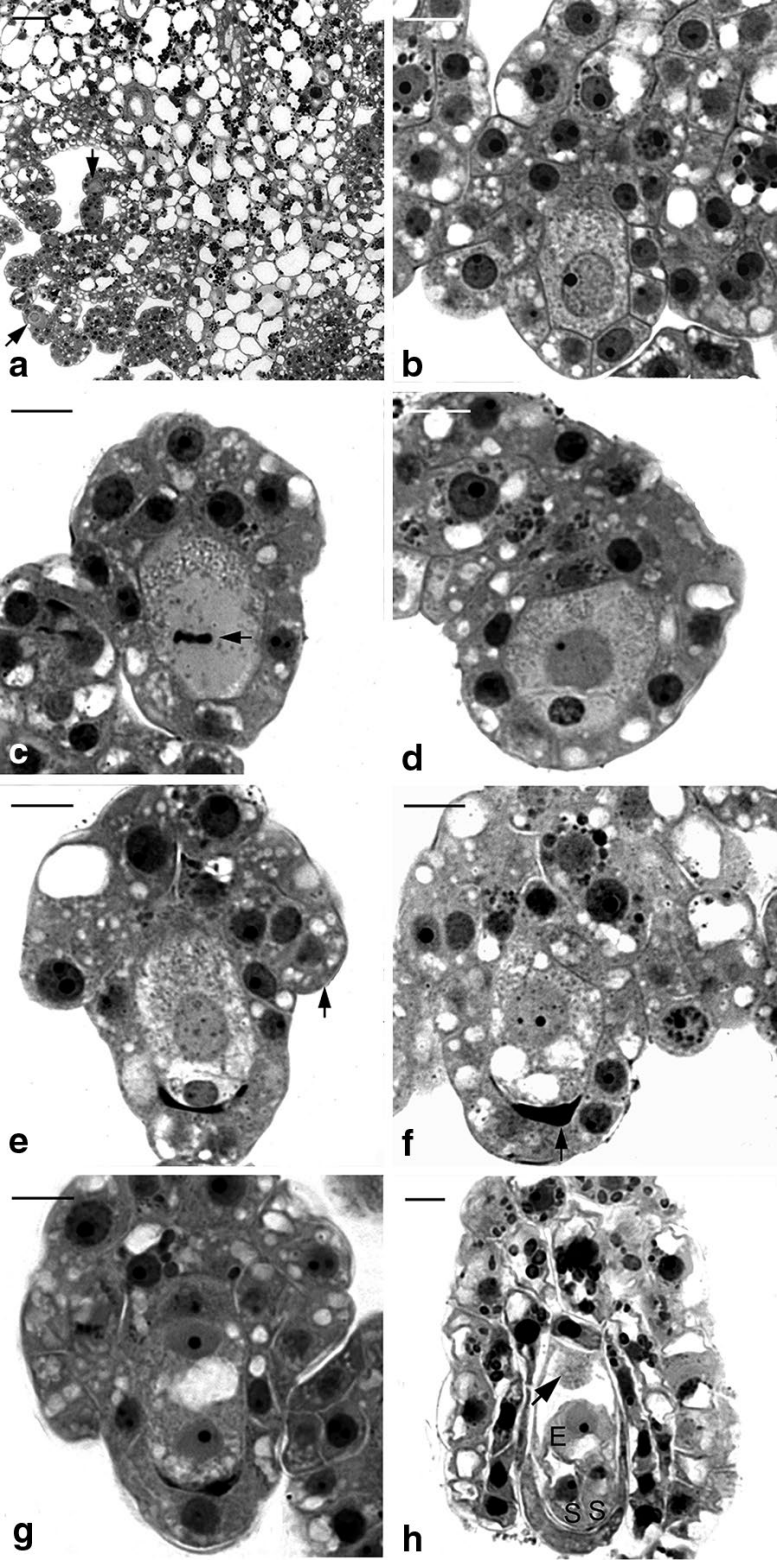
Light micrographs of megasporogenesis and megagametogenesis of *G. elata*. **a** Light micrograph showing the placental ridges with branches differentiated into nucellar filaments at the time of anthesis. The formation of archesporial cell (*arrows*) occurs at the terminus of the nucellar filament. *Scale bar* 50 μm. **b** The archesporial cell enlarges and differentiates into the megasporocyte. *Scale bar* 10 μm. **c** The megasporocyte undergoes the first meiotic division (*arrow*). *Scale bar* 10 μm. **d** After the first meiotic division, the functional dyad is located at the chalazal end of the ovule, and the non-functional dyad at the micropylar end degenerates gradually. *Scale bar* 10 μm. **e** After the second meiotic division, the smaller non-functional megaspore at the micropylar end degenerates soon. At the same time, the initiation of integument tissue has become visible (*arrow*). *Scale bar* 10 μm. **f** Light micrograph showing a functional megaspore with a few vacuoles and the non-functional megaspores have degenerated completely (*arrow*). *Scale bar* 10 μm. **g** The functional megaspore divides once resulting in the formation of a two-nucleate embryo sac, and the two-nucleate embryo sac expands further by vacuolation. *Scale bar* 10 μm. **h** At 4 DAP, a mature embryo sac showing one antipodal nucleus (*arrow*) and the egg apparatus, including the egg cell *E* and two synergids *S*. The integument tissue has not enclosed the embryo sac. *Scale bar* 10 μm

**Fig. 5:**
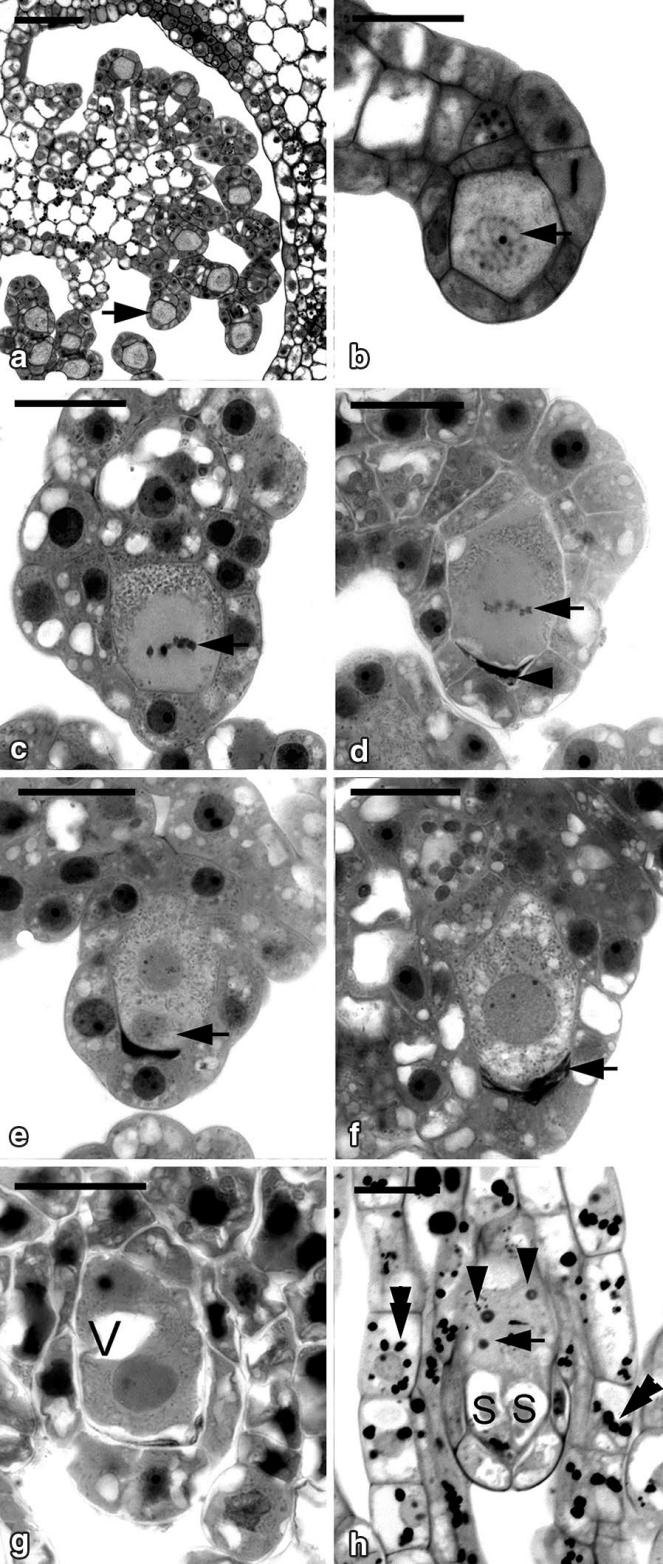
Light micrographs of megasporogenesis and megagametogenesis of *G. nantoensis*. **a** Light micrograph showing the placental ridges with several branches differentiated into nucellar filaments at the time of anthesis. The archesporial cell (*arrow*) can be observed at the terminus of the nucellar filament. *Scale bar* 50 μm. **b** The archesporial cell has differentiated into the megasporocyte. Before the first meiotic division, the chromatin (*arrow*) is going to condense. *Scale bar* 20 μm. **c** Soon, the megasporocyte undergoes the first meiotic division (*arrow*). *Scale bar* 20 μm. **d** After the first meiotic division, the functional dyad at the chalazal end of the ovule successively undergoes the second meiotic division (*arrow*), and the non-functional dyad at the micropylar end has degenerated (*arrowhead*). *Scale bar* 20 μm. **e** The second meiotic division results in the formation two megaspores of unequal size. The smaller non-functional megaspore (*arrow*) at the micropylar end will degenerate. *Scale bar* 20 μm. **f** Light micrograph showing a functional megaspore with several small vacuoles and the non-functional megaspores have degenerated completely (*arrow*). *Scale bar* 20 μm. **g** Light micrograph showing the two-nucleate embryo sac with a prominent vacuole (*V*) located at the center of cell. *Scale bar* 20 μm. **h** At 6 DAP, a longitudinal section through a mature embryo sac showing the egg apparatus, including the egg cell (*arrow*) and two synergids (*S*) at the micropylar end and two antipodal nuclei at the chalazal end (*arrowheads*). At this stage, the integument has completely enclosed the embryo sac. Several starch grains (*double arrowheads*) start to accumulate in the integument tissue. *Scale bar* 20 μm

In Orchidaceae, one of the unique features of ovule development is that mature ovules are usually not present at the time of pollination (Wirth and Withner 1959). The ovule development triggered by a successful pollination usually takes several weeks to complete (Arditti 1992, Yeung and Law 1997). In *G. elata* and *G. nantoensis*, at the time of anthesis, the placenta tissues were highly branched and the tip of a nucellar filament had differentiated into an archesporial cell (Figs. 4a, 5a). Prior to pollination, the archesporial cell had enlarged and differentiated into the megasporocyte (Figs. 4b, 5b). As Vij and Sharma (1986) pointed out, at the time of anthesis, three major types of ovule development could be observed in orchids. These types include (1) the ovule development has not been initiated prior to pollination, such as in *Cymbidium* (Swamy 1942), *Dendrobium* (Swamy 1943), *Epidendrum* (Yeung and Law 1989), *Phalaenopsis* (Duncan and Curtis 1942a; Zhang and O’Neill 1993); (2) the ovules contain well differentiated archesporial cells or megasporocytes, such as in *Cypripedium* (Duncan and Curtis 1942b; Law and Yeung 1993) and *Paphiopedilum* (Duncan and Curtis 1942b; Lee and Yeung 2012); (3) the mature ovules are ready for fertilization, such as in *Epipogium aphyllum* (Afzelius 1954), *Epipactis papillosa* (Sato 1974) and *G. elata* (Kusano 1915). In this study, it is clear that only the archesporial cell and/or megasporocyte could be observed within the ovaries of *G. elata* and *G. nantoensis* at the time of anthesis. The developmental stage of ovule in *Gastrodia* species at the time of anthesis is similar to those in slipper orchids, e.g. *Cypripedium* and *Paphiopedilum* (Law and Yeung 1993; Lee and Yeung 2012). However, the seed maturity in several *Cypripedium* and *Paphiopedilum* species usually takes 6–12 months. The faster seed development in *Gastrodia* species is not attributed to the presence of mature embryo sac, but the speedy courses of ovule and embryo development.

The changes in cell dimensions during embryo sac development of *G. elata* and *G. nantoensis* were summarized in Table 2. After pollination, the megasporocyte soon underwent the first meiotic division (Figs. 4c, 5c). The first meiotic division produced an unequal dyad: a smaller micropylar dyad and a larger chalazal dyad (Fig. 4d). The functional dyad at the chalazal end enlarged and performed the second meiotic division, while the micropylar dyad had become highly compressed (Fig. 5d). The second meiotic division resulted in the formation of two megaspores with unequal size: a smaller micropylar megaspore and a larger chalazal megaspore (Figs. 4e, 5e). The chalazal megaspore continued to enlarge and served as the functional megaspore, while the micropylar megaspore had compressed and become a thin dark-staining layer (Figs. 4f, 5f). The functional megaspore divided further and formed a two-nucleate embryo sac. The embryo sac had enlarged in size through the process of vacuolation at the center of the cell (Figs. 4g, 5g). In the mature embryo sacs of *G. elata* and *G. nantoensis*, four- to five-nucleate embryo sacs could be observed. Within the embryo sac, the egg apparatus containing one egg cell and two synergids was located at the micropylar end (Figs. 4h, 5h). In the central cell, one polar nucleus existed. At the chalazal pole, the antipodal nuclei were usually absent or occasionally one antipodal nucleus existed (Fig. 5h). The reduction of in the number of nuclei within the embryo sac has been reported as the ‘striking phenomenon’ (Harling 1950). This phenomenon has been observed in the ovule development of several orchids (Abe 1976; Arekal and Karanth 1981; Law and Yeung 1993). The reduction in the number of nuclei within the embryo sac may be due to the anomalies during the cell division process, such as the failure of mitosis, the fusion of mitotic spindles or the degeneration of existing chalazal nuclei (Yeung and Law 1997).

**Table 2:** Changes in cell dimensions during embryo sac development of *G. elata* and *G. nantoensis*

Developmental stage	Taxon	X-axis^a^	Y-axis^a^
Megasporocyte	*G. elata*	21.7 ± 0.7	29.4 ± 1.3
	*G. nantoensis*	20.4 ± 0.6	29.1 ± 1.5
Functional dyad	*G. elata*	20.8 ± 1.0	26.6 ± 1.7
	*G. nantoensis*	20.5 ± 0.8	28.7 ± 1.1
2-nucleate embryo sac	*G. elata*	25.1 ± 1.7	35.7 ± 2.5
	*G. nantoensis*	26.4 ± 1.1	36.1 ± 2.2
4-nucleate embryo sac	*G. elata*	28.5 ± 1.2	42.8 ± 1.8
	*G. nantoensis*	27.3 ± 1.7	44.6 ± 2.6
Mature embryo sac	*G. elata*	31.0 ± 1.1	53.1 ± 2.4
	*G. nantoensis*	31.7 ± 1.5	54.6 ± 1.9

^a^X, Y standard deviation in µm

### Embryo development

**Fig. 6:**
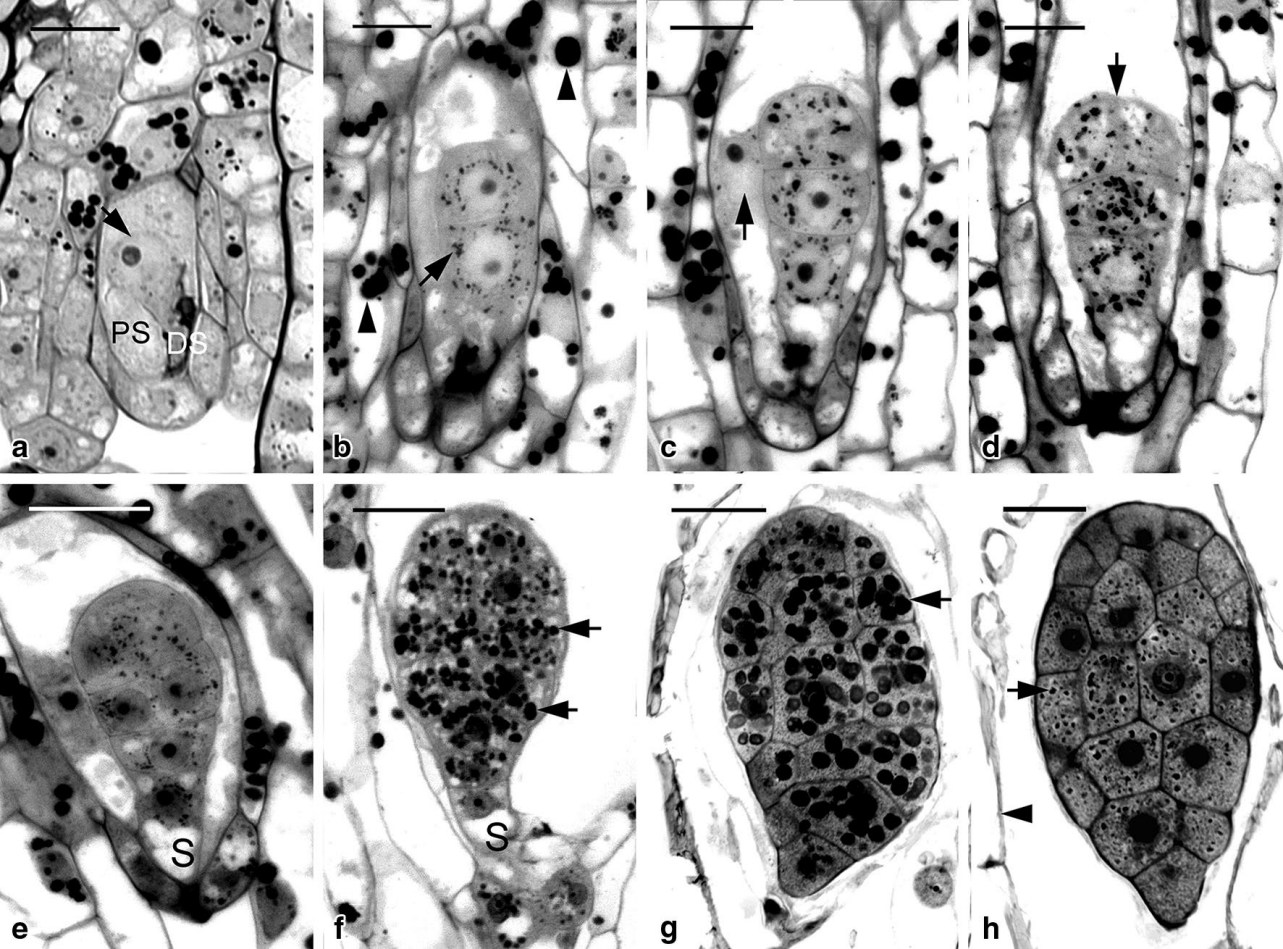
Light micrographs of embryo development of *G. elata*. **a** Light micrograph of the zygote after fertilization at 6 DAP. The zygote (*arrow*) is highly polarized with a chalazally located nucleus. At the micropylar end, one synergid has degenerated (DS), and the other synergid is still persistent (PS). *Scale bar* 20 μm. **b** The first cell division of the zygote results in the formation of a two-celled embryo. A few tiny starch grains (*arrow*) have appeared within the cytoplasm, and several larger starch grains (*arrowheads*) start to accumulate in the seed coat. *Scale bar* 20 μm. **c** An additional transverse division results in the formation of a three-celled embryo. The primary endosperm nucleus (*arrow*) could be observed within the endosperm cavity, but the endosperm eventually fails to develop. *Scale bar* 20 μm. **d** An anticlinal division (*arrow*) occurring in the terminal cell results in the formation of a four-celled embryo. *Scale bar* 20 μm. **e** Light micrograph showing a proembryo with a highly vacuolated suspensor cell (*S*). *Scale bar* 30 μm. **f** Light micrograph showing an early globular embryo with a single-celled suspensor (*S*). A large number of starch grains (*arrows*) accumulate within the cells of embryo proper. *Scale bar* 30 μm. **g** As the globular embryo approaches maturity, the size of starch grains (*arrow*) within the embryo proper has become larger and the suspensor finally degenerates. *Scale bar* 30 μm. **h** A longitudinal section through a mature seed at 16 DAP. At this stage, the starch grains have disappeared, and a number of small protein bodies (*arrow*) can be seen within the embryo proper. Although the lipid cannot be preserved in the historesin, the spaces between the protein bodies could be the storage lipid bodies. At maturity, the embryo is enveloped by a shriveled seed coat (*arrowhead*). *Scale bar* 30 μm

**Fig. 7:**
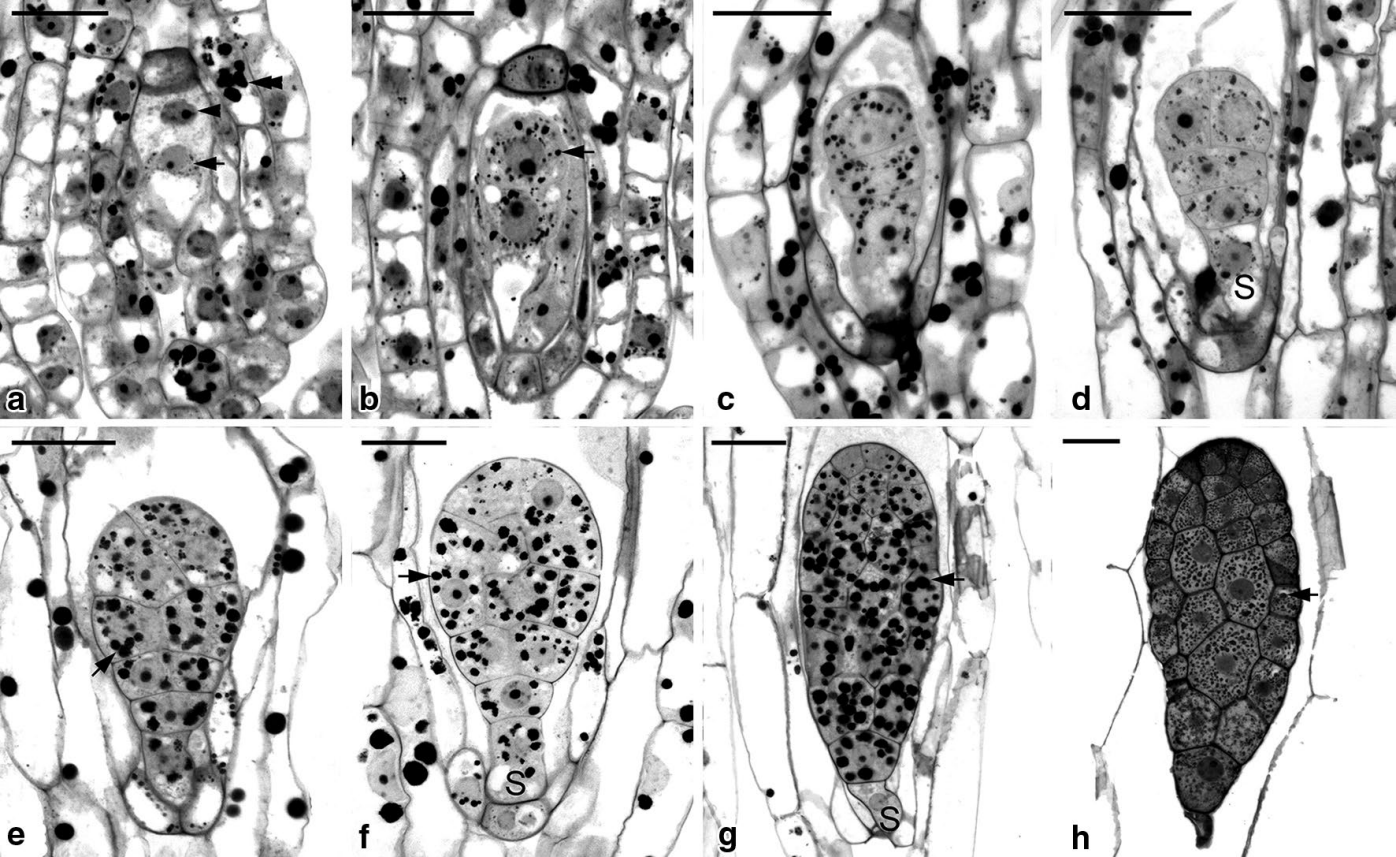
Light micrographs of embryo development of *G. nantoensis*. **a** Light micrograph of the zygote after fertilization at 8 DAP. The zygote (*arrow*) is highly polarized with a chalazally located nucleus and a prominent vacuole occupying the micropylar end. The polar-chalazal complex (*arrowhead*) includes the chalazal nuclei and the polar nuclei, but the endosperm eventually fails to develop. A number of starch grains (*double arrowhead*) could be seen in the seed coat. *Scale bar* 30 μm. **b** The first cell division of the zygote is unequal and results in the formation of a smaller terminal cell and a larger basal cell. Several tiny starch grains (*arrow*) have appeared within the cytoplasm. *Scale bar* 30 μm. **c** Light micrograph showing a three-celled embryo. The basal cell of the three-celled embryo will differentiate into the suspensor. *Scale bar* 30 μm. **d** Light micrograph showing a proembryo with a highly vacuolated suspensor cell (*S*). *Scale bar* 30 μm. **e** Light micrograph showing an early globular embryo. The size of starch grains (*arrow*) within the embryo proper becomes larger. *Scale bar* 30 μm. **f** A longitudinal section through a developing globular embryo. Large starch grains (*arrow*) are abundant and tend to congregate around the nucleus. The suspensor cell (*S*). *Scale bar* 30 μm. **g** As the seed approaches maturity, the cell division within embryo proper has ceased. At this stage, starch grains (*arrow*) are prominent within the embryo cells. The suspensor cell (*S*) has reduced its size and begins to degenerate. *Scale bar* 30 μm. **h** A longitudinal section through a mature seed at 20 DAP. The embryo has the smaller cells near the chalazal end and the larger cells in the micropylar end. At this stage, the starch grains have disappeared and numerous tiny protein bodies (*arrow*) have accumulated within the embryo proper. In this preparation, the lipid cannot be preserved, however the spaces between the protein bodies could be the storage lipid bodies. At maturity, the suspensor has degenerated and the embryo proper is enveloped by a shriveled seed coat. *Scale bar* 30 μm

Most of the ovules had been fertilized at 6 DAP in *G. elata* and at 8 DAP in *G. nantoensis* respectively, and the embryo development commenced (Table 1). The zygote was highly polarized with the nucleus located toward the chalazal end (Figs. 6a, 7a). In both species, their endosperms failed to develop. Within the endosperm cavity, the endosperm nuclei and the chalazal nuclei usually fused to a complex structure and it was eventually absorbed by the developing embryo. The first division of the zygote was unequal, giving rise to two daughter cells: a smaller terminal cell and a larger basal cell (Figs. 6b, 7b). The additional transverse cell division resulted in the formation of a three-celled embryo (Figs. 6c, 7c). The basal cell toward the micropylar end would become the suspensor, and the upper cells gave rise to the embryo proper. The suspensor of both species consisted of a single cell that was vacuolated with starch grains accumulated (Figs. 6e, 7e). Throughout the embryo development, no further division or enlargement was observed in the suspensor. As the embryo approached maturity, the suspensor became highly compressed and degenerated (Figs. 6h, 7h).

In *G. elata*, an anticlinal divisions in the terminal cell of the three-celled embryo resulted in the formation of a four-celled embryo (Fig. 6d), and in *G. nantoensis*, the occurrences of an anticlinal division in the terminal cell and transverse division in the subterminal cell of the three-celled embryo gave rise to a five-celled embryo (Fig. 7d). Additional divisions in the inner cell tiers of embryo proper resulted in the formation of an early globular embryo (Figs. 6e, 7e). Further anticlinal and periclinal cell divisions in the inner cell tiers of embryo proper gave rise to a globular embryo (Figs. 6f, 7f). Meanwhile, a large amount of starch grains accumulated in the developing embryo proper. As the embryo approached maturity (Figs. 6g, 7g), cell division had ceased within the embryo proper, and the accumulation of large starch grains was prominent. The composition and structure of these large starch grains remained to be investigated. At maturity, the embryo of *G. elata* took on an ovoid shape and was only seven cells long and four cells wide (Fig. 6h), while in *G. nantoensis*, the embryo had an ellipsoidal shape and was eight cells long and four cells wide (Fig. 7h). Like the mature seeds of many orchids, in *G. elata* and *G. nantoensis*, most starch grains had disappeared; instead, protein bodies and lipid bodies became the major storage products in their embryos (Yam et al. 2002). It is noteworthy that the mature embryos of *G. elata* and *G. nantoensis* consisted of a gradient of small to large cells (Figs. 6h, 7h). The marked gradient of cell size in the embryo proper can be observed in the easy-to-germinate species, such as *Phalaenopsis amabilis* var. *formosa* (Lee et al. 2008). On the contrary, in the difficult-to-germinate species, such as *Calypso bulbosa* (Yeung and Law 1992) and *C. formosanum* (Lee et al. 2005), no marked gradient of cell size within the embryo proper is observed. The presence of marked gradient of cell size within the globular embryo in orchids may speed the germination process. In the asymbiotic cultures, mature seeds of *G. elata* and *G. nantoensis* could readily germinate and reached the average germination percentage over 80 %.

**Fig. 8:**
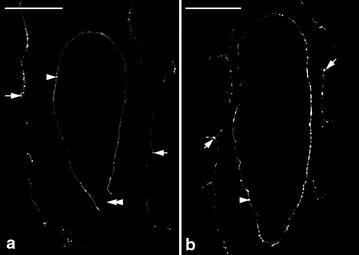
Nile red staining fluorescence micrograph of developing embryos of *G. nantoensis*. **a** Nile red staining fluorescence micrograph of a late globular embryo at the same stage as that seen in Fig. 7g. The innermost walls of the seed coat (*arrows*) and the surface wall (*arrowhead*) of the embryo proper react positively to the stain. The fluorescence is absent in the suspensor wall (*double arrowhead*). *Scale bar* 50 μm. **b** Nile red staining fluorescence micrograph of a mature seed at the same stage as that seen in Fig. 7h. The seed coat (*arrow*) and the surface wall (*arrowhead*) of the embryo proper react positively to the stain. *Scale bar* 50 μm

Nile red staining indicated that the cuticular material was deposited in the surface wall of embryo proper and the lateral walls of suspensor cell at the globular stage (Fig. 8a). As the seed matured, the accumulation of cuticular material was observed on the surface wall of embryo proper (Fig. 8b). Since the embryo of *Gastrodia* is housed within a thin layer of seed coat without the endosperm development, the deposition of cuticular materials in the surface wall of embryo proper could protect the globular embryo from early desiccation. The suspensor is a short-lived embryonic structure, and it is responsible for nutrient uptake for the developing embryo (Yeung and Meink 1993; Kawashima and Goldberg 2010). In orchids, the diverse of their suspensors provides distinguishing characteristics for a classification scheme for orchid embryo development (Swamy 1949). *G. elata* and *G. nantoensis* had a simple single-celled suspensor which belong to the type I group (Figs. 6f, 7f). It is remarkable that only the basal wall connecting to the nucellus reacts negatively to Nile red stain, while the lateral walls of suspensor react positively to Nile red stain (Fig. 8a), indicating that there is no cuticular material accumulated in the basal wall. The suspensor connects the embryo proper to the maternal tissues, and there are no direct symplastic connections between these two compartments. Therefore, in *Gastrodia*, the suspensor serves as the major conduit to supply the nutrients for the developing embryo proper as previously reported by Lee et al. (2006) and Lee and Yeung (2010).

### Seed coat development

In most orchids, their ovules are covered by the outer and inner integuments (known as bitegmic) (Law and Yeung 1993; Mayer et al. 2011), while in *G. elata* and *G. nantoensis*, their seed coats developed from a single integument (known as unitegmic), which were composed of two-celled layers (Figs. 6c, 7c). Species with simple ovule structures, e.g. the unitegmic ovule and the lack of a distinct micropyle are common features in some mycoheterotrophic orchids (Tohda 1967; Abe 1976). It has been proposed that the unitegmic ovules might be more advanced than the bitegmic ovules (Abe 1972). After fertilization, the cells of seed coat elongated rapidly and enclosed the embryo sac entirely. The cells of seedcoat were highly vacuolated and contained several starch grains (Figs. 6a, 7a). At maturity, the seed coat became dehydrated and compressed into a thin layer that enveloped the embryo (Figs. 6h, 7h). In the developing seed of *G. nantoensis*, the outermost wall of seed coat gave a weak fluorescence from Nile red staining, suggesting the presence of little cuticular material (Fig. 8a, b). However, the weak fluorescence in the seed coat can be easily quenched by pre-staining of sections with TBO, indicating that a distinct cuticle may be absent (see Holloway 1982; Yeung et al. 1996). TBO is a cationic dye that binds to negatively charged groups in cells and gives two main spectra of reaction, i.e. pinkish purple for carboxylated polysaccharides such as pectin and greenish blue for aromatic substances such as lignin (Pradhan Mitra and Loqué 2014). Under fluorescence microscopy, if TBO staining causes the fluorescence quenching of the cell wall by UV-excitation, we would suggest the accumulation of lignin rather than cuticular material in the cell wall. In this study, the compressed seed coat stained greenish blue with TBO, indicating the lignified cell wall of the seed coat (Figs. 6h, 7h). In orchids, the lignified seed coat could protect the minute globular embryo at the time of seed dispersal.

## Conclusions

The present study illustrates the key anatomical events in the seed development of *G. elata* and *G. nantoensis* and identifies a number of features that are not yet described clearly in the previous studies, such as the initial differentiation of ovule at the time of anthesis, the histochemical characters of the cell wall of suspensor and seed coat, and the changes of storage products within the embryo proper. This study provides basic knowledge for further molecular studies on embryo development and symbiotic germination of *Gastrodia* species.
